# Synthesis and the impact of hydroxyapatite nanoparticles on the viability and activity of rhizobacteria

**DOI:** 10.3762/bjnano.16.17

**Published:** 2025-02-18

**Authors:** Bedah Rupaedah, Indrika Novella, Atiek Rostika Noviyanti, Diana Rakhmawaty Eddy, Anna Safarrida, Abdul Hapid, Zhafira Amila Haqqa, Suryana Suryana, Irwan Kurnia, Fathiyah Inayatirrahmi

**Affiliations:** 1 Research Center for Applied Microbiology, National Research and Innovation Agency, Bogor, Indonesia; 2 Mathematics and Natural Science Faculty, Padjadjaran University, Jatinangor, Indonesia; 3 Research Center for Mining Technology, National Research and Innovation Agency, Lampung, Indonesia; 4 Chemical Engineering Department, University of Indonesia, Depok, Indonesia

**Keywords:** biofertilizer, carrier material, nanohydroxyapatite, rhizobacteria

## Abstract

Preserving the viability of rhizobacteria during plant application poses a significant challenge when utilizing rhizobacteria as biofertilizers, especially under adverse environmental conditions. Therefore, the selection of a suitable carrier material for rhizobacteria plays a crucial role in ensuring the sustained viability of these microorganisms. Nanomaterials, particularly nanohydroxyapatite (nHA), have garnered attention for sustaining rhizobacterial viability, high loading capacity, high biodegradability, and biocompatibility, which facilitate microbial interactions. In this study, nHA was synthesized using a hydrothermal method and used as a carrier for two rhizobacteria strains (Pd and Tb). The structural and morphological properties of nHA were examined through XRD and scanning electron microscopy analyses. Rhizobacteria were encapsulated within the carrier material, and their viability was evaluated using the total plate count method. Following their immobilization on nHA, the phosphate-solubilizing capacity of rhizobacteria was evaluated using Pikovskaya’s medium. A nitrogen-free bromothymol medium was utilized to qualitatively assess the nitrogen-fixing ability of rhizobacteria. Furthermore, rhizobacteria were identified using 16S rRNA gene sequencing, followed by analysis to construct a phylogenetic tree. nHA was found to meet the required quality criteria, exhibiting a spherical morphology with an average particle size of 68 nm and a porosity of 54.78%. The nHA carrier demonstrated favorable physical attributes to sustaining rhizobacterial viability with pH 8.95 and an electrical conductivity of 55.4 μS/cm. Rhizobacteria loaded onto the nHA carrier maintained comparable viability to those without carriers. The highest viability of the rhizobacterial strains Pd and Tb loaded onto the nHA carrier was observed on the seventh day after inoculation, measuring at 2.480 × 10^7^ and 1.040 × 10^7^ CFU/mL, respectively. The qualitative tests of nHA as rhizobacterial carrier demonstrated that rhizobacteria retained their ability to solubilize phosphate and fix nitrogen. Furthermore, both rhizobacteria have been identified. Pd rhizobacterium was identified with complete match to *Brevundimonas olei* strain Prd2. Similarly, Tb rhizobacterium showed 100% similarity to *Bacillus altitudinis* strain NPB34b. Based on this reseach, nanohydroxyapatite could be the potential carrier to protect rhizobacteria from external stressors and to maintain their viability over the long term. These findings indicate the potential of a nanohydroxyapatite–rhizobacteria system as a promising environmentally friendly fertilizer.

## Introduction

In recent years, Indonesia has observed a troubling and progressive increase in land degradation, primarily caused by the excessive use of chemical fertilizers. This issue is compounded by the growing population and the subsequent increase in food demands, leading to widespread consequences such as the loss of soil biodiversity, pollution of both soil and water, and disruption of the natural ecosystem balance [1]. To tackle these pressing environmental challenges, a promising solution lies in adopting biofertilizers in agriculture, which involve harnessing microorganisms like plant growth promoting rhizobacteria. These microorganisms possess the potential to profoundly benefit plant growth, yield, and overall productivity. Moreover, they play a crucial role in bolstering plant resistance to pathogens, thereby enhancing plant resilience [2–3].

Preserving the viability of rhizobacteria during plant application presents a significant challenge when utilizing rhizobacteria as biofertilizers, particularly under adverse environmental conditions. Selecting an appropriate carrier material for rhizobacteria ensures their sustained viability. The primary function of the carrier material is to protect rhizobacteria from external stressors and maintain their viability over time [4]. Consequently, the characteristics of the carrier material used to transport rhizobacteria are critical in determining their survival and successful colonization within both soil and root systems. This, in turn, contributes to the development of biofertilizers as a viable alternative to reduce the dependence on chemical fertilizers [5].

In recent years, there has been a notable increase in interest regarding the utilization of nanomaterials as carrier materials. Nanometer-sized carriers offer a substantial surface area and demonstrate exceptional compatibility, making them ideal candidates for serving as carrier materials [6]. Additionally, nanoscale carrier materials offer the advantage of high loading capacity, remarkable stability, and the ability to enhance rhizobacterial resilience against drought conditions. Moreover, they provide convenience in both application and storage [7]. Nanomaterials have gained significant attention in the development of rhizobacterial carrier materials, as their effective utilization can provide protective benefits to plants, assist in nutrient absorption, and, when in gel form, significantly improve water management efficiency. Furthermore, they require only small quantities for utilization [8].

Several types of nanoparticles have been employed as carriers for rhizobacteria inoculants, including silica nanoparticles [9], clay nanoparticles [8], carbon nanoparticles [10], zinc oxide nanoparticles [11], and calcium carbonate nanoparticles [12]. Among these, nanohydroxyapatite (nHA) offers distinct advantages over other nanoparticles regarding the application as a carrier for rhizobacteria, particularly in terms of phosphorus supply [13], biological compatibility [14], and high adsorption capacity [15]. Consequently, nHA not only enhances the survival of rhizobacteria but also promotes plant growth by providing essential nutrients.

nHA emerges as a remarkable biomaterial widely embraced as a nanocarrier, primarily because of its porous structure that facilitates precise and efficient conduction and release of various materials [16]. nHA serves as a versatile agent in drug delivery [17], acts as a carrier for genes and proteins [18], and aids in immobilizing rhizobacteria for effective heavy metal removal [19]. In addition to its carrier capabilities, nHA exhibits exceptional attributes such as high biodegradability, biocompatibility, and the ability to absorb organic substances [20–21]. Furthermore, nHA possesses the unique ability to be resorbed within physiological environments while remaining non-toxic, a feature that holds significant promise for synergistic interactions with microorganisms and biological molecules [22–23].

Modern research endeavors are marked by a range of pioneering developments regarding hydroxyapatite (HA), aimed at creating highly effective HA nanoparticles customized for the use as carrier materials. These nanoparticles are undergoing thorough examination as carriers for rhizobacteria, capitalizing on the unique porous nature and architecture of the material. These architectures can function as protective habitats, safeguarding microorganisms from various challenges, and providing colonization opportunities within the pore spaces [4,24].

This study endeavors to investigate the efficacy and efficiency of hydrothermally synthesized nHA as a specialized carrier material for rhizobacterial strains, including Pd and Tb rhizobacteria. The results of this research have the potential to enhance the efficacy of biofertilizers formulated with nanoparticles as carriers with the aim to enhance plant productivity.

## Results and Discussion

### Synthesis of nanohydroxyapatite

The synthesis of nHA was effectively achieved through a hydrothermal method, conducted at a temperature of 230 °C for a duration of 48 h. The resultant nHA material was obtained in the form of white powder. The formation of nHA occurred gradually through a solvothermal reaction, driven by the chemical transformation represented by the following equation:


[1] $$\begin{array}{l} 10\text{Ca}{\left(\text{OH}\right)}_{2\left(\text{aq}\right)}+6{\left({\text{NH}}_{4}\right)}_{2}{\text{HPO}}_{4\left(\text{aq}\right)}\to \\ {\text{Ca}}_{10}{\left({\text{PO}}_{4}\right)}_{6}{\left(\text{OH}\right)}_{2\left(\text{s}\right)}↓+12{\text{NH}}_{4}{\text{OH}}_{\left(\text{aq}\right)}+6{\text{H}}_{2}\text{O.} \end{array}$$

### Nanohydroxyapatite characterization

**Figure 1:**
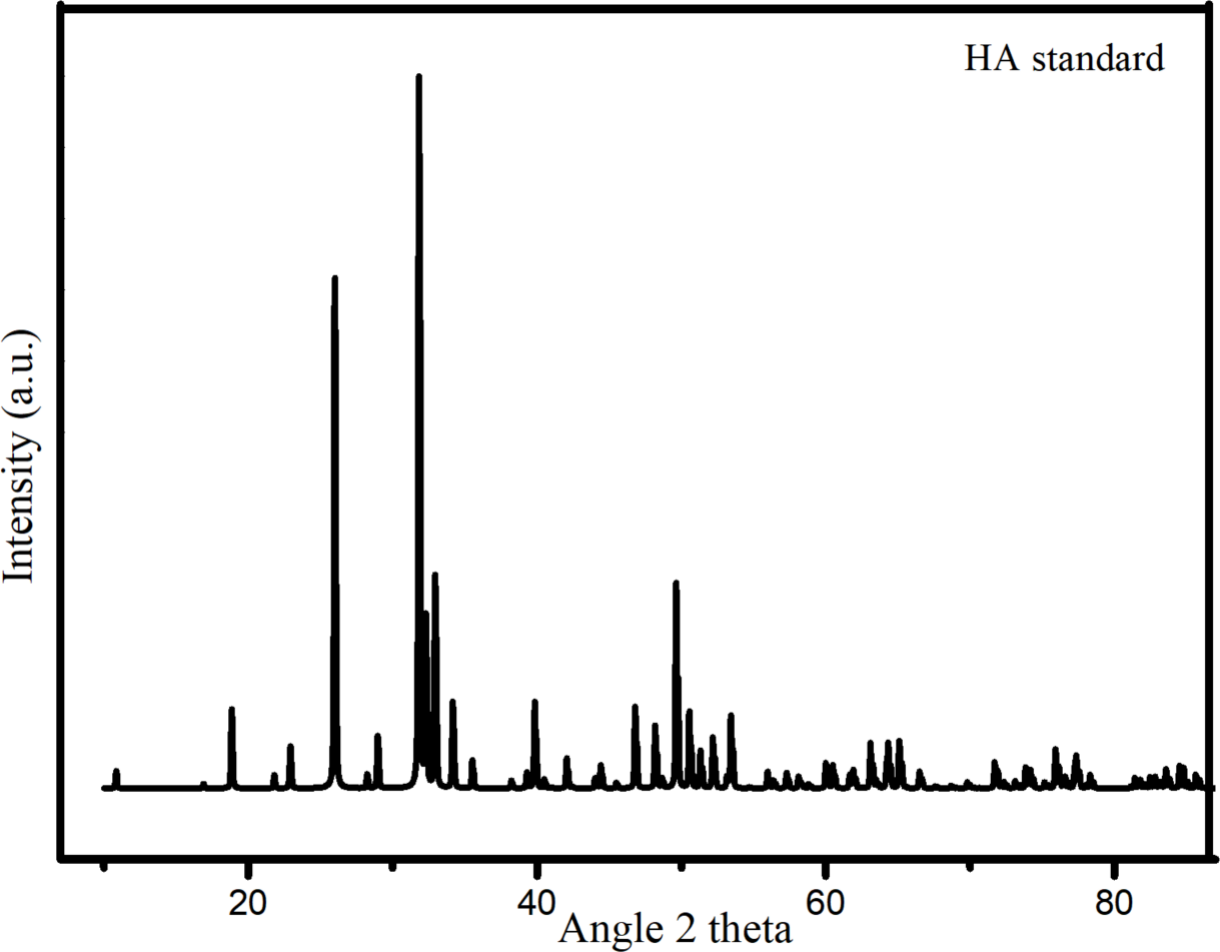
XRD pattern of HA standard (ICSD #157481).

**Figure 2:**
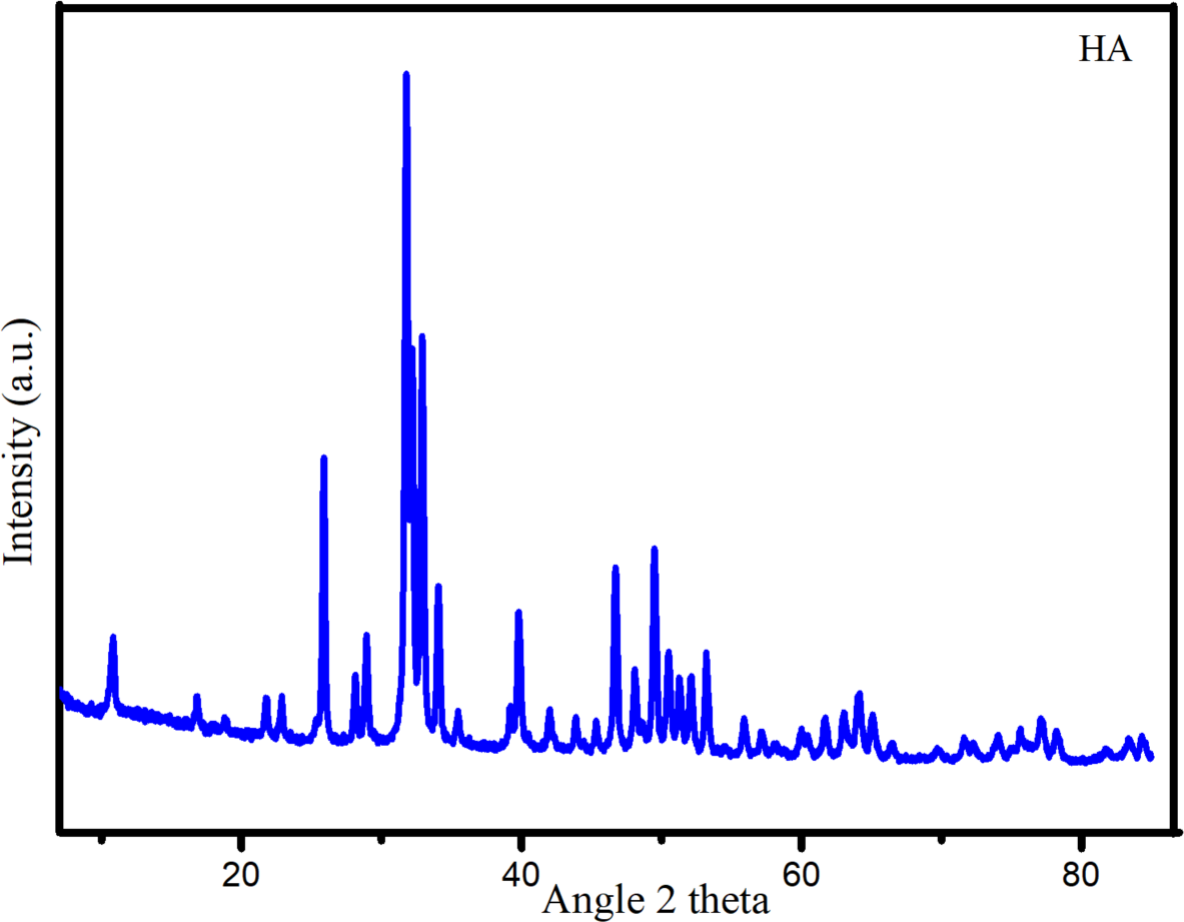
XRD pattern of the synthesized nHA.

The structural analysis of nHA was performed using X-ray diffraction (XRD). The diffraction pattern of nHA standard is given in Figure 1, while that of the synthesized nHA is given in Figure 2.

Figure 2 shows that the XRD pattern of nHA aligns with the ICSD #157481 standard (Figure 1) and the *P*6_3_/*m* space group. This alignment confirms the successful synthesis of hydroxyapatite. Notably, the image highlights the characteristic (211) peak of HA at 2θ = 31.77°, which is the highest peak of HA, as reported by Noviyanti et al. [25] and Novella and coworkers [26]. Using the information obtained from the XRD pattern, the crystal size (*D*) of the sample can be calculated utilizing the Scherrer equation, as outlined by Monshi and coworkers [27]:


[2] $$D=\frac{K\lambda }{\beta \cos\theta },$$

where *K* is a constant (with a value of 0.89), λ represents the X-ray wavelength in nanometers (0.154 nm), β denotes the full width at half maximum (FWHM) of the diffraction peak, and θ is the Bragg angle. The crystal size of the sample was found to be 34.27 nm, with a full width at half maximum (FWHM) of 0.2384 radians.

**Figure 3:**
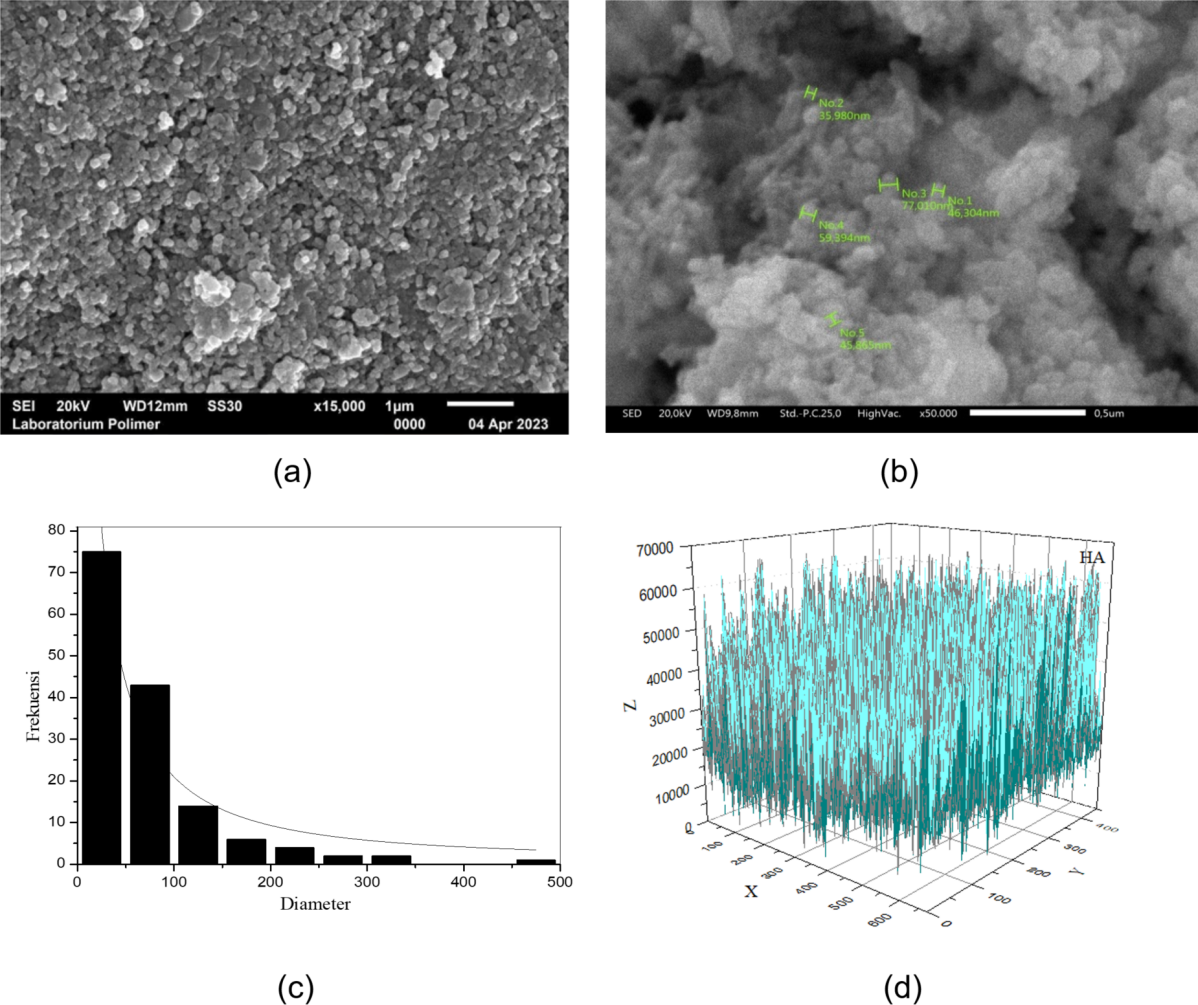
Morphology and particle size distribution of the sample, (a, b) SEM images at magnifications of 15,000× and 50,000×, (c) particle size distribution, and (d) 3D plot of porosity.

The morphology of the analyzed sample, observed through scanning electron microscopy (SEM) at magnifications of 15,000× and 50,000× are depicted in Figure 3.

Figure 3 provides a clear view of the sample demonstrating spherical shapes with a consistent particle size distribution. The SEM analysis results reveal an average particle size of 68.08 nm, as displayed in Figure 3b. Moreover, a more comprehensive examination of the SEM image using OriginLab software yields a sample porosity of 54.78%, as illustrated in Figure 3d. In this representation, the blue regions correspond to the solid volume of the material, while the white areas represent the voids or pores within the material. For an overview of the characteristics of the nHA carrier, please refer to Table 1.

**Table 1:** Characteristics of the nHA carrier.

Parameter	nHA carrier^a^

pH	8.95
EC (μs/cm)	55.4

^a^The data represents the average of two measurements.

The pH and EC values are important factors that influence the psychochemical properties of a material. Changes in pH and EC values can affect the stability of a substance, ion interactions, and molecular ionization in a solution. However, the effect of pH and EC values on the psychochemical properties highly depends on the material’s application [28–29]. Pd rhizobacterium typically thrives optimally at pH 7.5 and exhibits resilience against pH fluctuations ranging from 5.5 to 9 [30]. Similarly, Tb rhizobacterium shows its best growth within a pH range of 7 to 8 [31].

### Viability of rhizobacteria-nHA

**Figure 4:**
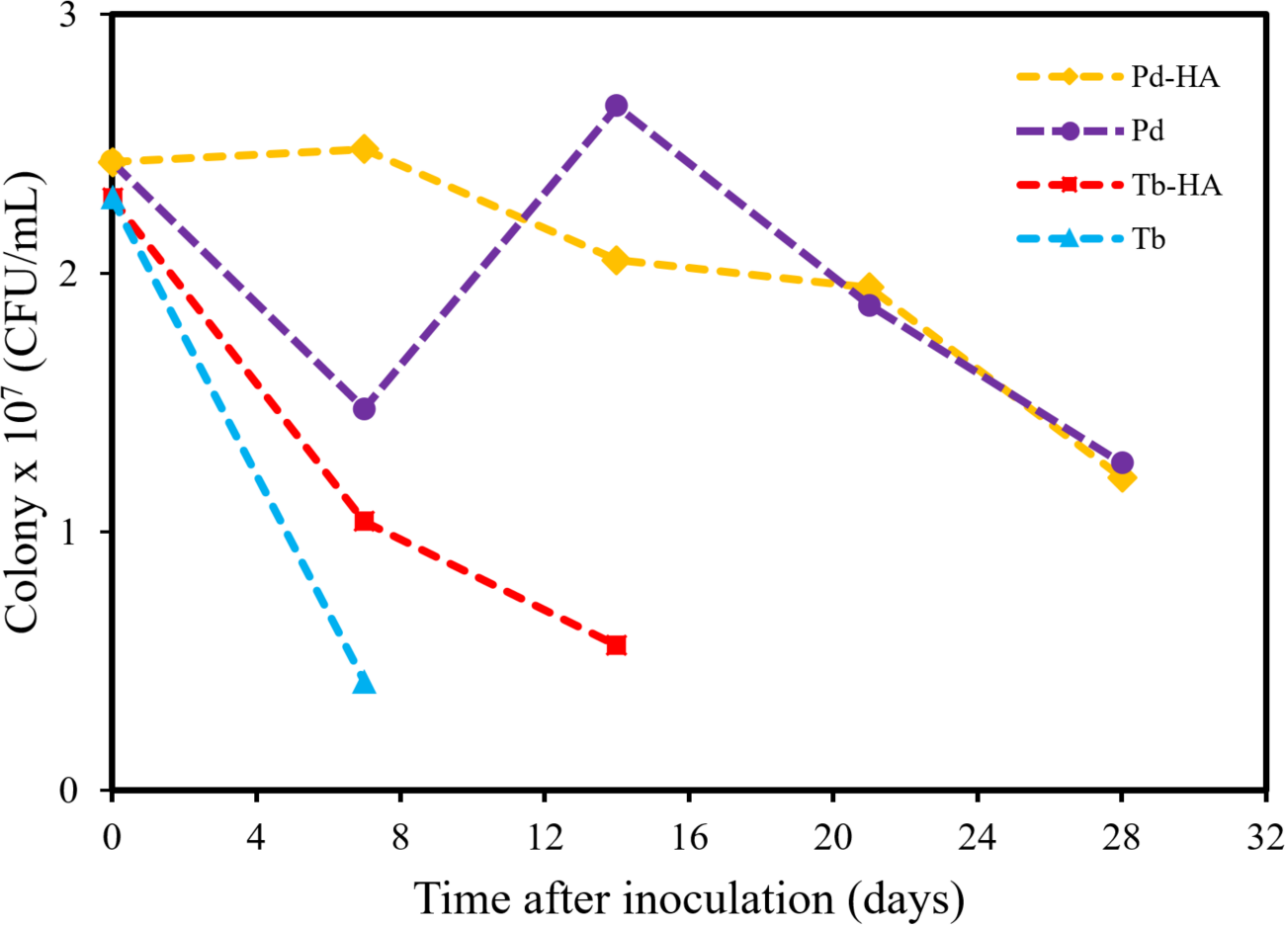
Viability of rhizobacteria on the nHA carrier after 28 days of incubation.

The viability test of rhizobacteria loaded onto the nHA carrier and incubated for 28 days yielded significant findings. Notably, Pd and Tb rhizobacteria reached their highest population counts seven days after inoculation. Specifically, Pd rhizobacterium achieved a peak count of 2.48 × 10^7^ CFU/mL on the nHA carrier. Similarly, Tb rhizobacterium exhibited a population of 1.04 × 10^7^ CFU/mL on the nHA carrier after seven days. Interestingly, the population of Pd rhizobacterium on the nHA carrier declined over the observation period, whereas no Tb rhizobacteria were detected after seven days of nHA carrier inoculation. Based on the data presented in Table 2 and Figure 4, rhizobacteria loaded onto the carrier demonstrate a comparable level of survivability to rhizobacteria without carrier. Moreover, the viability of Tb rhizobacterium loaded onto the carrier is notably higher compared to rhizobacteria without carrier. These findings suggest that nHA can effectively serve as a carrier for preserving rhizobacteria intended for plant applications.

**Table 2:** The number of rhizobacteria colonies quantified using the total plate count (TPC) method.

Rhizobacterial strains	Carrier	Number of colonies (CFU/mL)				
		day				

		0	7	14	21	28

Pd rhizobacterium	nHA	2.43 ×10^7^	2.48 × 10^7^	2.05 × 10^7^	1.95 × 10^7^	1.21 × 10^7^
	without carrier	2.43 × 10^7^	1.48 × 10^7^	2.65 × 10^7^	1.88 × 10^7^	1.27 × 10^7^
Tb rhizobacterium	nHA	2.29 × 10^7^	1.04 × 10^7^	0.56 × 10^7^	—	—
	without carrier	2.29 × 10^7^	0.42 × 10^7^	—	—	—

The nHA carrier is hydroxyapatite (Ca_10_(PO_4_)_6_(OH)_2_) in powder form, comprising the elements calcium, phosphorous, oxygen, and hydrogen. These elements provide nutrients that support the viability of rhizobacteria. Water within the carrier also plays a crucial role in maintaining the viability of rhizobacteria [32].

Figure 4 illustrates that the Tb rhizobacterium strain does not maintain viability on the carrier 14 days after inoculation. However, compared to rhizobacteria without a carrier, the presence of the carrier enhances viability on the seventh day after inoculation.

**Figure 5:**
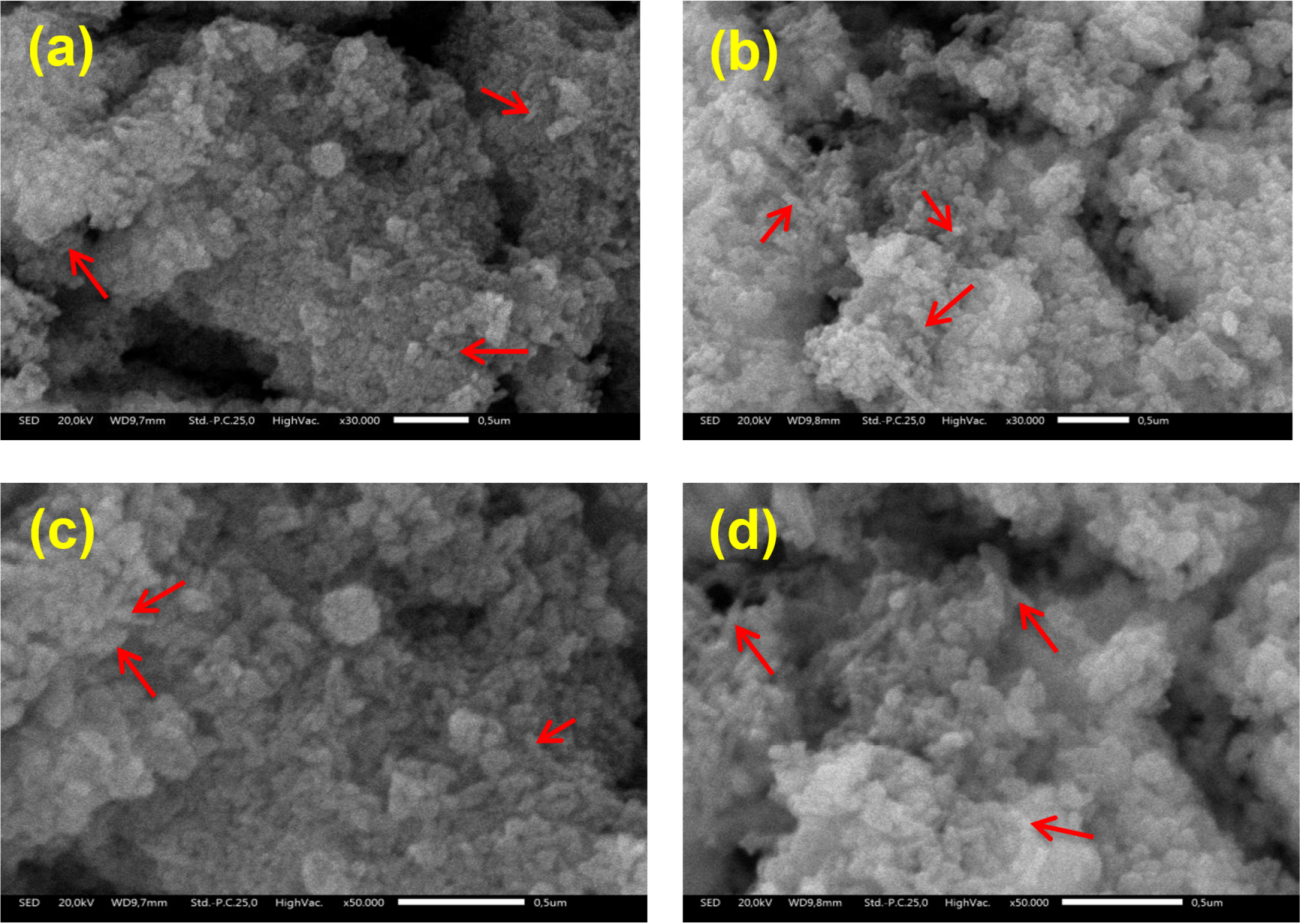
SEM images of rhizobacteria loaded onto nHA. (a) nHA-Pd at 30,000× magnification, (b) nHA-Tb at 30,000× magnification, (c) nHA-Pd at 50,000× magnification, and (d) nHA-Tb at 50,000× magnification.

Distinct morphological differences are evident between rhizobacteria and the nHA carrier material. The nHA carrier material exhibits spherical shapes, whereas Pd and Tb rhizobacteria are rod-shaped (shown by red arrows). The morphological features observed in nHA are depicted in Figure 5. The mechanism of rhizobacterial attachment to the nHA carrier material is not fully understood. However, the structure of nHA, characterized by its voids and crevices, provides an environment conducive to the growth and survival of rhizobacteria, a phenomenon known as biological fixation [33].

**Figure 6:**
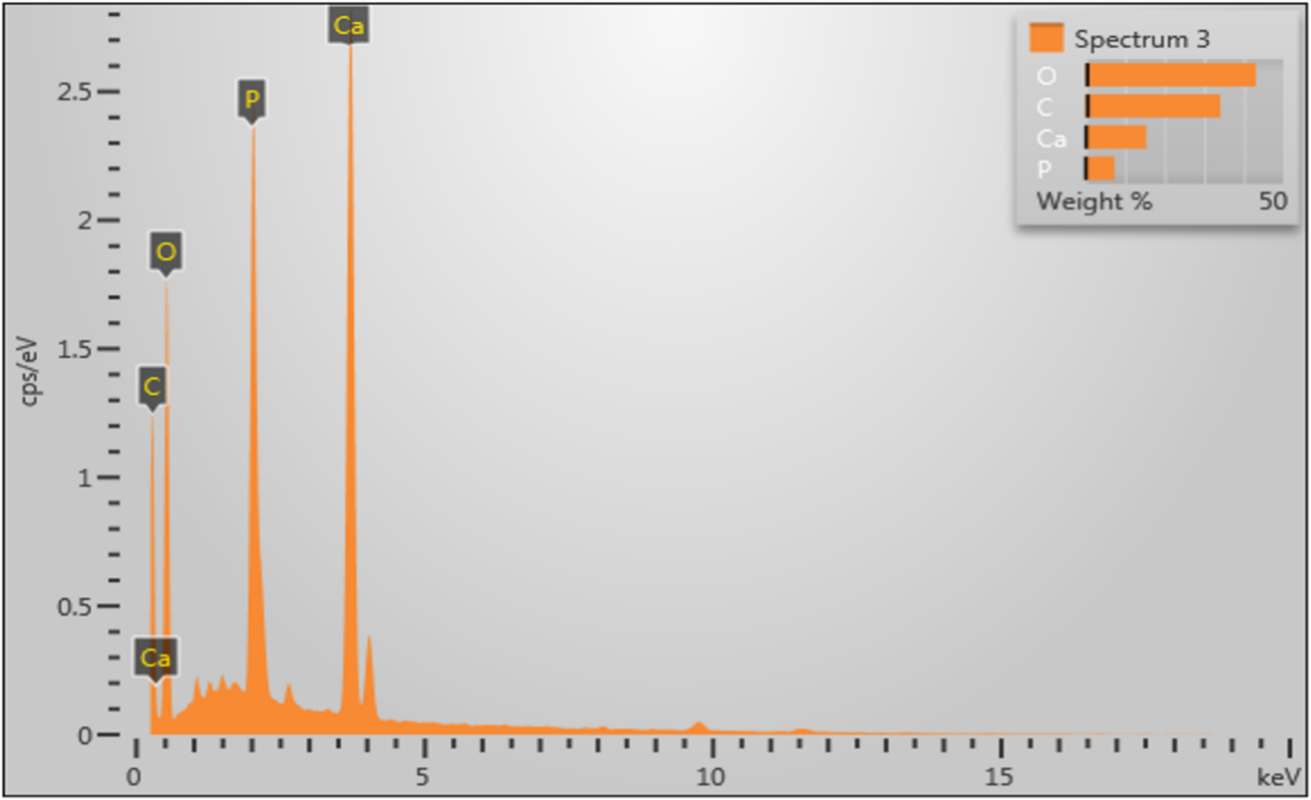
EDX images of rhizobacteria loaded onto nHA. (a) Pd-nHA and (b) Tb-nHA.

Figure 6, along with Table 3 and Table 4, shows the elemental composition of the rhizobacteria-nHA comprising Ca, P, O, and C. Ca, P, and O constitute the components of the nHA carrier material, while C originates from the rhizobacteria. The percentage of carbon in the Pd-nHA sample is notably higher, reaching 59.04%, compared to the 46.14% of the Tb-nHA sample. These findings correlate with the higher colony count of the Pd rhizobacterium strain as outlined in Table 2.

**Table 3:** Elemental composition of the Pd-nHA sample.

Element	wt %	wt % sigma	atom %	Standard label

C	47.75	0.58	59.04	C Vit
O	37.79	0.56	35.09	SiO_2_
P	4.72	0.11	2.26	GaP
Ca	9.74	0.16	3.61	wollastonite
				
Total:	100.00		100.00	

**Table 4:** Elemental composition of the Tb-nHA sample.

Element	wt %	wt % sigma	atom %	Standard label

C	34.13	0.70	46.14	C Vit
O	43.12	0.63	43.76	SiO_2_
P	7.37	0.15	3.86	GaP
Ca	15.38	0.24	6.23	wollastonite
				
Total:	100.00		100.00	

### Phosphate-solubilizing capability of rhizobacteria-nHA

The proficiency of rhizobacteria in phosphate solubilization after inoculation on the nHA carrier for 21 days underscores their preserved capability, as evidenced by the emergence of clear zones. The presence of these clear zones in the Petri dishes denotes the compatibility of rhizobacterial strains with the utilized carrier, corroborating previous reports [34]. Table 5 provides the solubilizing index values for strains of Pd and Tb rhizobacteria. After 21 days of rhizobacterial inoculation on the carrier, there are minimal changes in the solubilizing index values compared to pre-inoculation levels. The Tb rhizobacterium strain loaded onto the nHA carrier exhibits a higher solubilizing index than its pre-loading state. Rhizobacteria demonstrate their ability to create transparent zones on Pikovskaya media because of their capability for phosphate solubilization. These microorganisms produce organic acids, such as citric acid and oxalic acid, which bind to phosphate via hydroxy groups and chelating cations such as Fe, Al, and Ca. Thus, soluble phosphate forms that plants can absorb are formed. The organic acids generated by rhizobacteria facilitate the formation of phosphate complexes, producing H_2_PO_4_^−^ and the subsequent appearance of clear zones within the medium [35–36].

**Table 5:** Solubilizing index (SI) of rhizobacteria loaded onto the nHA carrier.

Rhizobacteria strains	Carrier	Solubilizing index (SI)	

		initial	21 days after inoculation

Pd rhizobacterium	nHA	2.6917	2.3992
	without carrier	2.6917	2.5385

Tb rhizobacterium	nHA	2.4031	2.5583
	without carrier	2.4031	2.3140

### Nitrogen-fixing activity of rhizobacteria-nHA

**Figure 7:**
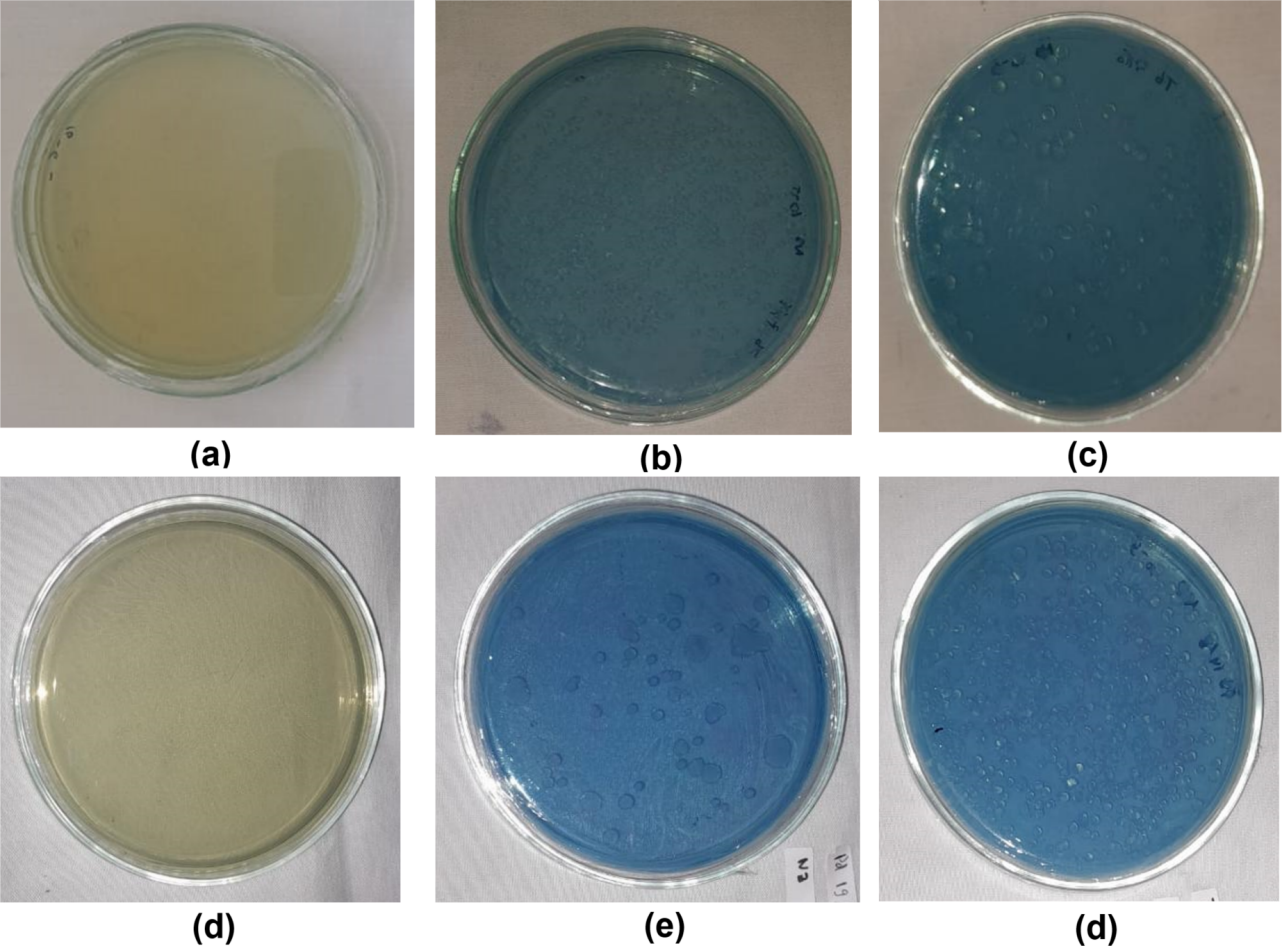
Nitrogen-fixing activity of rhizobacteria loaded onto the carrier after 28 days of incubation. (a) Control, (b) Pd rhizobacterium, (c) nHA-Pd, (d) control, (e) Tb rhizobacterium, and (f) nHA-Tb.

The nitrogen-fixing activity of rhizobacteria after loading onto the nHA carrier was evaluated qualitatively by monitoring the color transition in nitrogen-free bromothymol (NFB) medium from yellow to blue. The results presented in Table 6 and Figure 7 indicate that all rhizobacteria loaded onto the carrier exhibited nitrogen fixation.

**Table 6:** Nitrogen-fixing activity of rhizobacteria loaded onto the carrier.^a^

No.	Carrier	Pd rhizobacterium				Tb rhizobacterium			

		day				day			
		7	14	21	28	7	14	21	28

1.	nHA	+	+	+	+	+	+	+	+
2.	without carrier	+	+	+	+	+	+	+	+

^a^+ (positive): change in color; − (negative): no change in color.

### Molecular identification and morphological analysis of rhizobacteria

The 16S rRNA sequencing of Pd and Tb rhizobacterial strains had two stages of analysis. The first stage involved generating consensus sequences by combining the forward and reverse primary sequences. The second stage involved comparing these consensus sequences with those in the NCBI online library. At the first stage, the 16S rRNA sequence from the Pd rhizobacterium resulted in a consensus sequence of 1330 bp, and the 16S rRNA sequence from the Tb rhizobacterium yielded a consensus sequence of 1425 bp. The kinship between Pd and Tb rhizobacteria was analyzed using a phylogenetic approach, a classification method that considers the evolutionary paths of the organisms studied [37]. The phylogenetic tree of Pd and Tb rhizobacteria was reconstructed using the neighbor joining method. This method utilizes pairwise distance (p-distance) values to assess the closeness or distance of kinship between Pd and Tb rhizobacteria and ten reference species. The p-distance values were calculated using the two-parameter Kimura evolutionary model. The rationale behind this method is to use the smallest index of p-distance values between pairs of organisms to group them together. However, this method may result in trees with misaligned branch lengths between organisms as it assumes that the rate of evolution for the same gene among organisms is not uniform.

**Figure 8:**
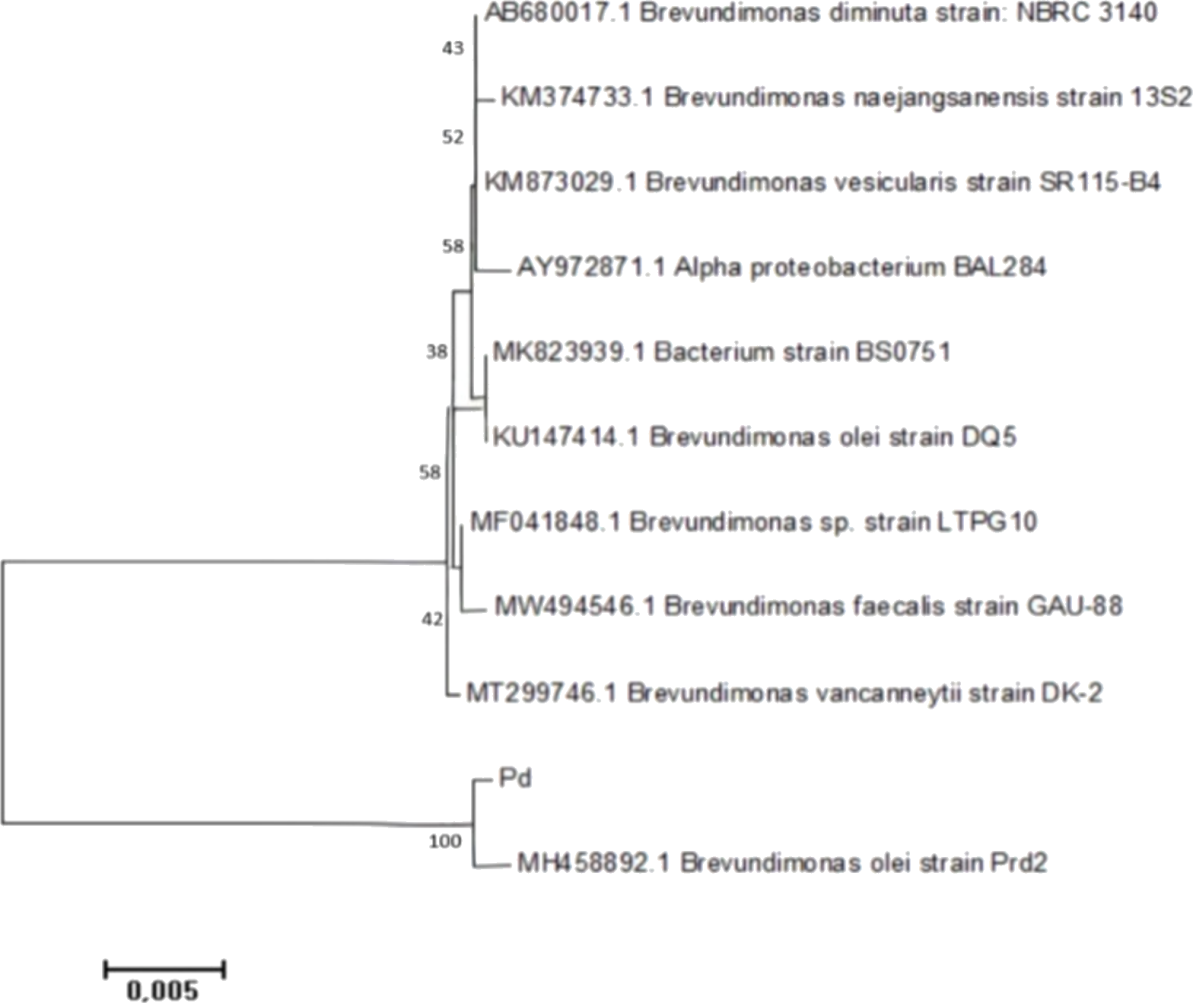
Phylogenetic tree of the Pd rhizobacterium based on the 16S rRNA gene.

**Figure 9:**
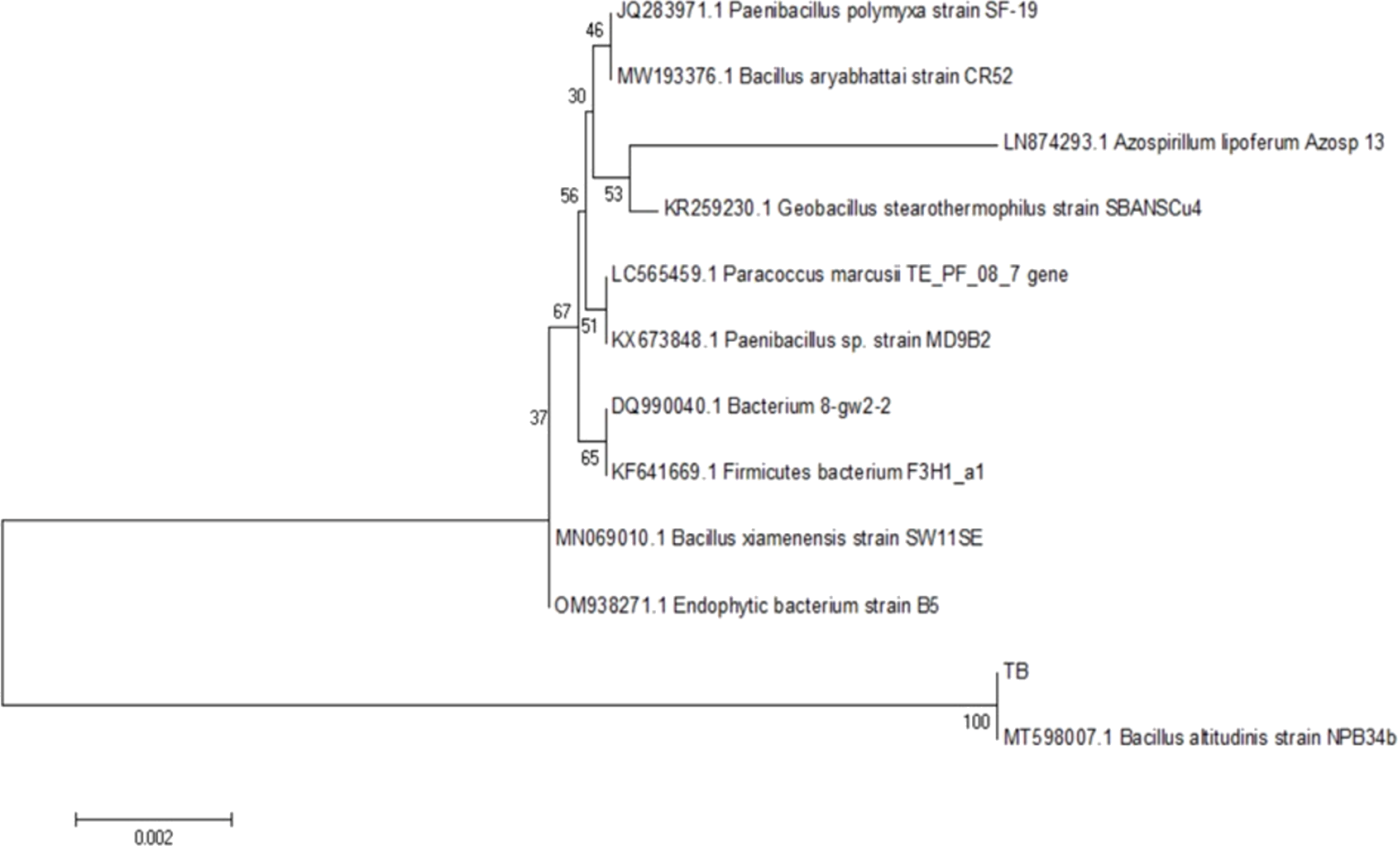
Phylogenetic tree of the Tb rhizobacterium based on the 16S rRNA gene.

The Pd rhizobacterium was identified as *Brevundimonas olei*, sharing 100% similarity with *Brevundimonas olei* strain Prd2 (Figure 8). Similarly, the Tb rhizobacterium was identified as *Bacillus altitudinis*, showing 100% similarity to *Bacillus altitudinis* strain NPB34b (Figure 9). According to Drancourt et al. [38] and Janda and Abbott [37], species are considered to have a similarity greater than 97%, with matching identification if the similarity exceeds 99%. A similarity lower than 97% might suggest the presence of a novel species, as indicated by Stackebrandt and Goebel [39].

**Figure 10:**
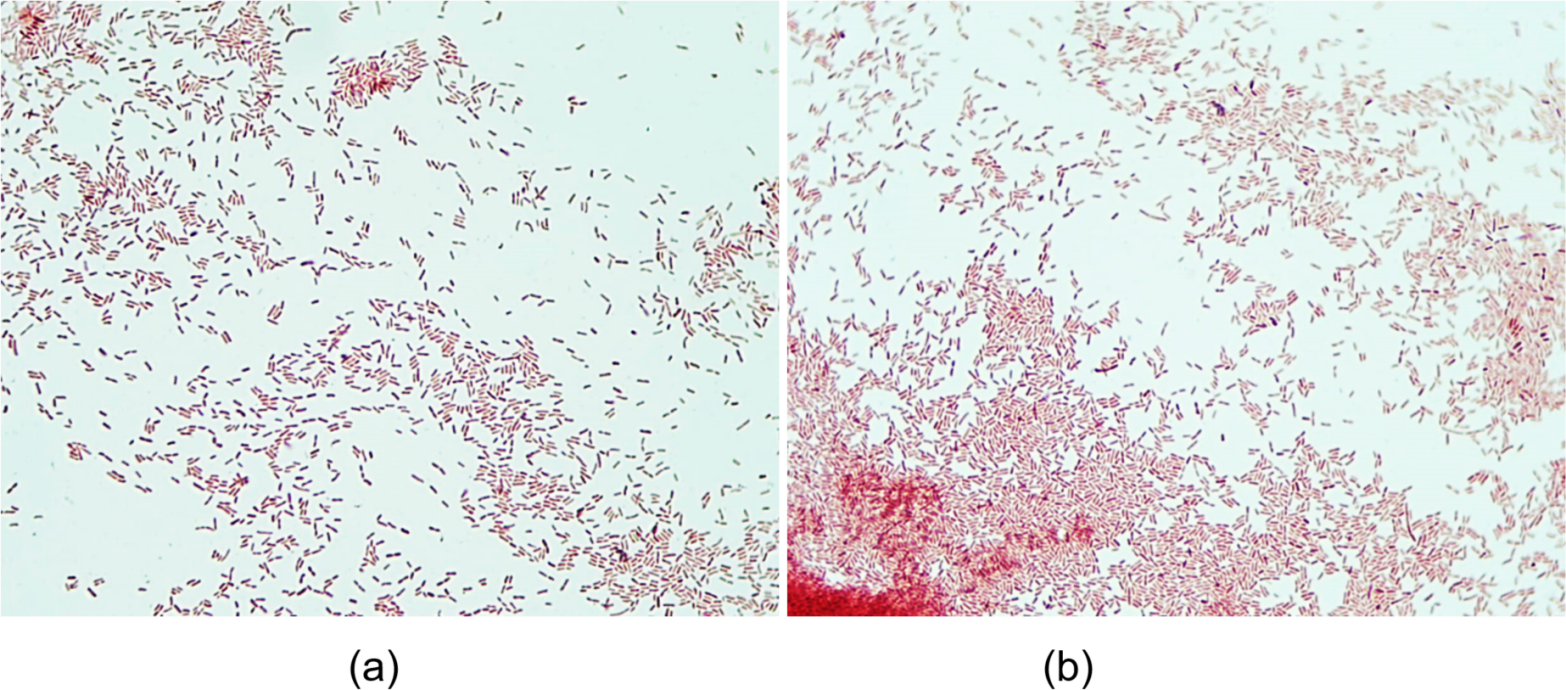
Rhizobacterial cell morphology. (a) *Brevundimonas olei* (Pd) and (b) *Bacillus altitudinis* (Tb).

Staining revealed that both *Brevundimonas olei* (Pd) and *Bacillus altitudinis* (Tb) are gram-positive rhizobacteria with rod-like morphology, as illustrated in Figure 10.

## Conclusion

Nanohydroxyapatite (nHA) was synthesized successfully using a hydrothermal method, and XRD analysis confirmed its alignment with the ICSD standard #154781. The measured crystal size of nHA was 34.27 nm. SEM analysis revealed a spherical shape with an average particle size of 68.08 nm and a porosity of 54.78%. Rhizobacteria loaded onto the nHA carrier exhibited viability comparable to rhizobacteria incubated without a carrier. Over the observation period, the viability of both types of rhizobacteria, Pd and Tb rhizobacteria, decreased. nHA utilized as a carrier for rhizobacteria demonstrated good compatibility, as the loaded rhizobacteria retained their phosphate-solubilization activity, evident from the appearance of clear zones. Additionally, the nitrogen-fixing activity was maintained as indicated by the color change in NFB medium. Two rhizobacterial strains have been successfully identified. The Pd rhizobacterium was identified as *Brevundimonas olei*, demonstrating 100% similarity to *Brevundimonas olei* strain Prd2; the Tb rhizobacterium was identified as *Bacillus altitudinis*, exhibiting 100% similarity to *Bacillus altitudinis* strain NPB34b.

## Experimental

### Nanohydroxyapatite synthesis

The synthesis of nHA in this study builds upon previous protocols. Specifically, a mixture containing 2.7925 g of calcium oxide and 3.9663 g of diammonium phosphate (with a Ca/P ratio of 1.67) was dissolved in 100 mL of distilled water. This solution was introduced into an autoclave and heated at 230 °C for 48 h. The resulting precipitate was carefully filtered, washed with deionized water until reaching a pH level of 7, and dried at 110 °C for 2 h, following the protocol established by Yusuf et al. [40] and Novella and coworkers [26].

### Nanohydroxyapatite characterization

Structural assessment of the synthesized nHA was conducted using X-ray diffraction (XRD) with the PANalytical AERIS system and OriginLab 8.5.1 software. Further examination of the sample’s morphology was carried out using a scanning electron microscope JSM-6510LA JEOL SEM, followed by in-depth analysis using ImageJ and OriginLab 8.5.1 software tools. Additionally, the physical parameters of nHA utilized as a carrier material, including pH and electrical conductivity (EC), were determined using a digital pH meter (Mettler Toledo, MP220 pH meter) and precise digital measurement of electrical conductivity (Mettler Toledo, MC 226 conductivity meter).

### Preparation of inoculum and formulation of rhizobacteria-nHA

nHA was carefully placed into 2 mL EP tubes and underwent sterilization through autoclaving. The rhizobacteria were introduced into sterilized Nutrient Broth (NB) media, which had undergone heat treatment at 120 °C for 20 min, followed by a 72 h incubation period on a shaker. Subsequently, nHA was inoculated with rhizobacterial suspension at a 1:1 (w/v) ratio. The resulting solid inoculum of rhizobacteria-nHA underwent incubation for specified time intervals (7, 14, 21, and 28 days) at a controlled temperature of 28 °C, following the methodologies outlined by Rakian et al. [41] and Safari and coworkers [7].

### Viability test of rhizobacteria-nHA

This study aimed to assess the viability of rhizobacteria encapsulated within the carrier material. The assessment utilized the total plate count (TPC) method, following the methodologies outlined by Sohaib and coworkers [34]. To conduct the experiments, formulations of rhizobacteria-nHA were initially suspended in sterile water at 10^−1^ dilution. Subsequently, 1 mL of the rhizobacteria-nHA suspension (10^−1^) was carefully transferred using a sterile pipette and diluted into 9 mL of sterile water. This mixture was homogenized through vortexing. This dilution process was performed for a range of dilutions, spanning from 10^−2^ to 10^−7^. Following the dilution steps, 0.1 mL of the 10^−5^, 10^−6^, and 10^−7^ dilutions were plated onto nitrogen-free bromothymol (NFB) medium. These plates were then incubated for a period of 14 days, after which colony counts were determined.

### Evaluating the phosphate-solubilizing capability of rhizobacteria-nHA

To assess the phosphate-solubilizing capacity of rhizobacteria immobilized on nHA, Pikoskaya medium was utilized, following the methodology outlined by Safari and coworkers [7]. Formulations of rhizobacteria-nHA were diluted with sterile water. Subsequently, 5 μL of the resulting suspension was introduced into each quadrant of the Pikoskaya medium, followed by incubation at 28 °C for 8 days. The qualitative evaluation of phosphate-solubilization ability was indicated by the formation of a clear zone within the growth media (Supporting Information File 1, Figure S2). Following the incubation period, measurements were taken of both the diameter of the clear zone (halo) and the bacterial colony. The solubilizing index (SI) was then calculated using the formula: SI = (colony diameter + clear zone diameter)/colony diameter.

### Assessment of nitrogen-fixing activity of rhizobacteria-nHA

The nitrogen-fixing ability of rhizobacteria was assessed qualitatively using a NFB medium [42]. This medium comprises K_2_HPO_4_, FeCl_3_·6H_2_O, MgSO_4_·7H_2_O, NaCl, CaCl_2_, MnSO_4_·7H_2_O, biotin, KOH, malic acid, bromothymol blue, Na_2_MoO_4_, and bacto agar. Rhizobacteria loaded onto nHA were introduced into the medium and left to incubate for 14 days. The nitrogen-fixing capability was determined by observing rhizobacterial growth and monitoring the color change of the medium from yellow to blue within the agar medium (Supporting Information File 1, Figure S1).

### Biological materials and molecular identification

The Pd and Tb rhizobacterial strains used in this study were obtained from the Research Center for Applied Microbiology, National Research and Innovation Agency. Both rhizobacterial strains have been characterized regarding their nitrogen fixation and phosphate-solubilizing abilities. To revive these rhizobacteria, the streaking method was applied on NFB medium, followed by an incubation process consisting of three sets, each lasting 24 h. For strain identification, DNA extraction was performed using Instagene™ Matrix, followed by sample amplification using 16S rRNA primers (5′-AGA GTT TGA TCC TGG CTC AG-3′) and (5′-GGA TAC CTT GTT ACG ACT T-3′), with a fragment length of 1500 base pairs. The primer concentration used was 10 pmol/μL. The PCR composition consisted of 12.5 μL GoTaq^®^ Green Master Mix, 2 μL of forward and reverse primers, 50 ng/μL DNA template, and 6.5 µL dH_2_O. The PCR cycle was carried out as follows: an initial denaturation at 95 °C for 5 min, followed by 30 cycles consisting of denaturation at 94 °C for 45 s, annealing at 52 °C for 1 min, and elongation at 72 °C for 1 min. After the final cycle, polymerization continued at 72 °C for 5 min. The PCR products were then analyzed by electrophoresis on a 1% agarose gel. Visualization was performed under UV light using a UV transilluminator. The purified DNA fragments were used as templates in sequence analysis. The PCR products of the 16S rDNA were labeled using the Big Dye Terminator Reaction Mixture Sequencing KIT from Perkin Elmer. The sequencing process was carried out by First Base and further analyzed using Clustal W and MEGA6 programs to construct a phylogenetic tree. BLAST analysis of all consensus sequences revealed high similarity and query coverage with 16S rRNA sequences on the NCBI database. Several similar sequences obtained from BLAST were downloaded and utilized for phylogenetic tree reconstruction and genetic distance analysis.

## Supporting Information

File 1 Activity tests of Pd and Tb rhizobacteria.

## Data Availability

Data generated and analyzed during this study is available from the corresponding author upon reasonable request.
