# Supplementary material for: Synthesis and the impact of hydroxyapatite nanoparticles on the viability and activity of rhizobacteria
Source: Beilstein J Nanotechnol. 2025 Feb 18;16:216–28. doi: 10.3762/bjnano.16.17 (PMC11849556; doi:10.3762/bjnano.16.17)
Supplement: File 1 — Activity tests of Pd and Tb rhizobacteria. [file Beilstein_J_Nanotechnol-16-216-s001.pdf]

BEILSTEIN JOURNAL OF NANOTECHNOLOGY

Supporting Information

for

Synthesis and the impact of hydroxyapatite nanoparticles on the viability and activity of rhizobacteria

Bedah Rupaedah, Indrika Novella, Atiek Rostika Noviyanti, Diana Rakhmawaty Eddy, Anna Safarrida, Abdul Hapid, Zhafira Amila Haqqa, Suryana Suryana, Irwan Kurnia and Fathiyah Inayatirrahmi

Beilstein J. Nanotechnol. **2025,** *16,* 216–228. doi:10.3762/bjnano.16.17

Activity tests of Pd and Tb rhizobacteria

License and Terms: This is a supporting information file under the terms of the Creative Commons Attribution License (https://creativecommons.org/licenses/by/4.0). Please note that the reuse, redistribution and reproduction in particular requires that the author(s) and source are credited and that individual graphics may be subject to special legal provisions.
The license is subject to the *Beilstein Journal of Nanotechnology* terms and conditions: (https://www.beilstein-journals.org/bjnano/terms)

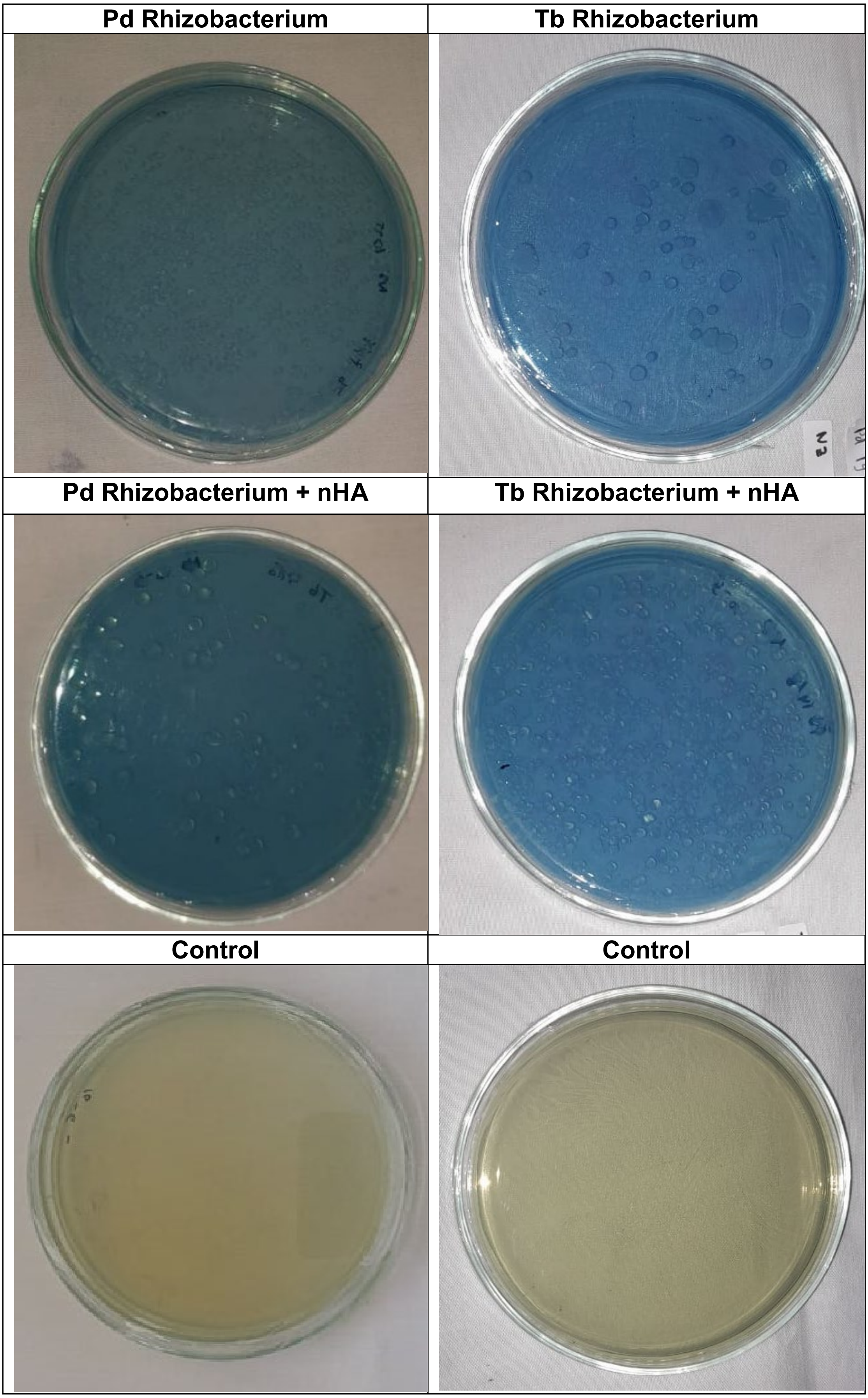

Figure S1: Nitrogen-fixation test of Pd and Tb rhizobacteria.

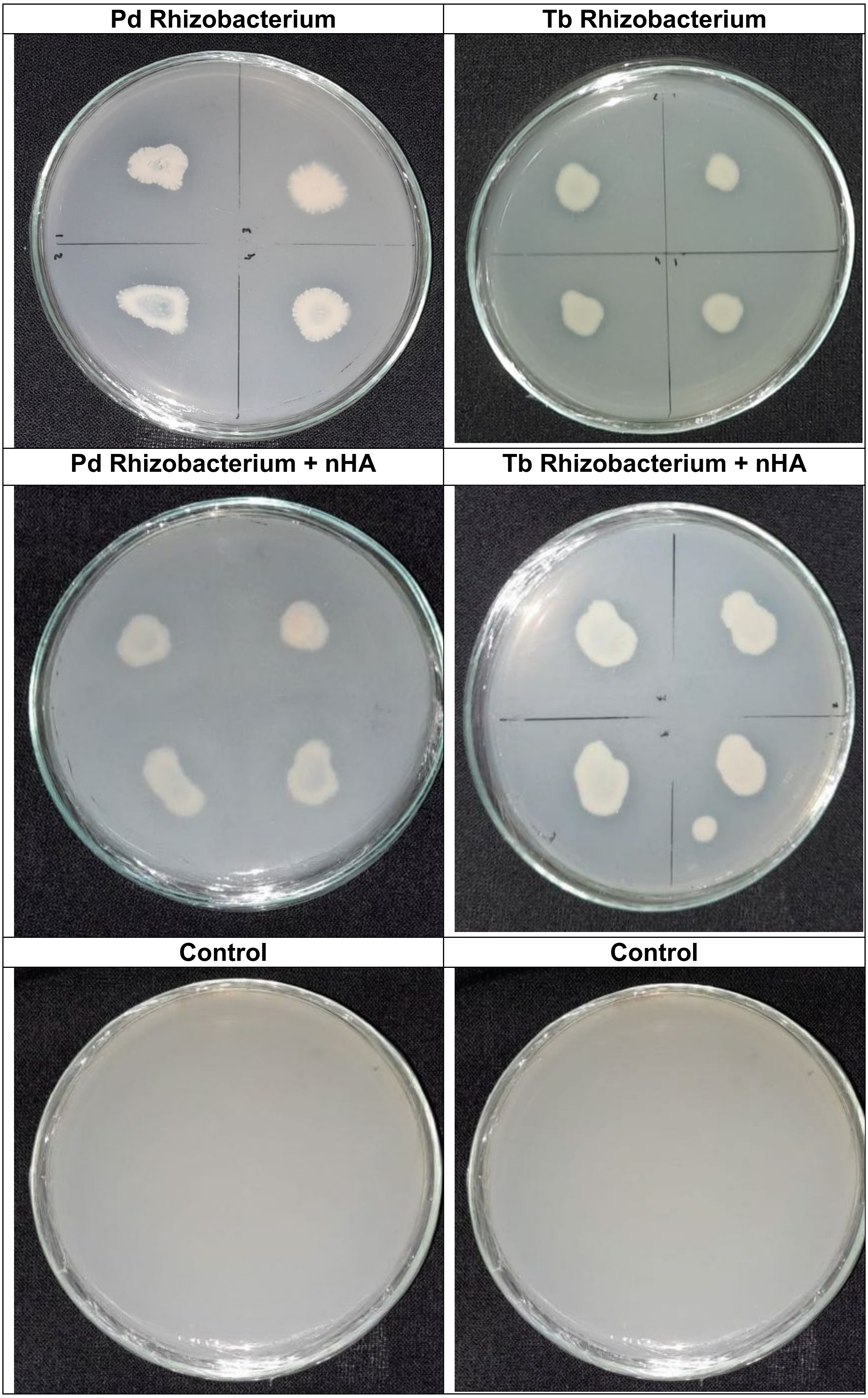

Figure S2: Phosphate-solubilizing test of Pd and Tb rhizobacteria.
